# Risk taking propensity: Nurse, surgeon and patient preferences for diverting ileostomy

**DOI:** 10.1111/codi.16149

**Published:** 2022-05-04

**Authors:** Ian Mackay, David A. Clark, James Nicholson, Aleks Edmundson, Daniel Steffens, Michael Solomon

**Affiliations:** ^1^ Royal Brisbane and Women's Hospital Brisbane Qld Australia; ^2^ Faculty of Medicine and Health, Central Clinical School The University of Sydney Sydney NSW Australia; ^3^ Surgical Outcomes Research Centre (SOuRCe) Royal Prince Alfred Hospital Sydney NSW Australia; ^4^ University of Qld Brisbane Qld Australia; ^5^ St Vincent's Private Hospital Northside Brisbane Qld Australia; ^6^ Salford Royal NHS Foundation Trust University Teaching Hospital Salford UK

**Keywords:** anastomotic leak, diverting ileostomy, nurse and patient personality, risk taking index, surgeon personality, willing to gamble, willing to trade

## Abstract

**Aim:**

The decision‐making process to defunction a pelvic colorectal anastomosis involves complex heuristics and is framed by surgeon personality factors. Risk taking propensity may be an important factor in these decisions and patient preferences have not been evaluated alongside surgeons and nurses.

**Methods:**

A prospective cross‐sectional study involving a one‐off interview and questionnaire assessing how risk taking propensity affects nurse, surgeon and patient preferences for a temporary defunctioning ileostomy (TDI) was performed. The risk taking index (RTI) was employed to evaluate risk taking propensity and the validated prospective measures of preference instruments to evaluate preferences for stoma avoidance in several scenarios by asking the individual to consider trading or gambling years of remaining life expectancy.

**Results:**

One hundred and fifty participants met the inclusion criteria, which included 30 (20.0%) surgical nurses, 20 (13.3%) colorectal surgeons and 100 (66.7%) patients. Surgeons had a significantly higher RTI (mean ± SD: 26.8 ± 6.7) than patients (mean ± SD: 20.0 ± 9.8) and nurses (mean ± SD: 23.0 ± 6.6) *p* = 0.002. Surgeons would consider that it would be in a patient's best interest to have a TDI at an AL rate of 15% or greater, whereas nurses and patients would do so at 28% and 25%, respectively (*p* = 0.007).

**Conclusion:**

Surgeons were shown to have a higher risk taking propensity than patients and nurses but a significantly lower threshold of AL where they would consider a TDI is in the best interest of the patient.

**What does this paper add to the literature?**

This study frames the decision to incorporate a diverting stoma with the individual's risk taking propensity and evaluates the preferences of surgeons alongside those of patients and nurses.

## INTRODUCTION

The decision‐making process to defunction a pelvic colorectal anastomosis involves complex heuristics (a mental shortcut, or “rule of thumb” to allow a person to make a decision quickly and is often based on past similar experiences) and is framed by surgeon personality factors [1]. The description and understanding of these factors is important to understand variations in clinical practice for similar clinical scenarios. In studies from 2014 and 2017, MacDermid et al. found that surgeons aged under 50 years of age and surgeons with a lower propensity for risk taking were independent predictors of stoma formation after anterior resection [2, 3]. Personality traits in colorectal surgeons were studied by Moug et al, and favourable personality traits for surgery were found and that they potentially influenced the next anastomotic decision [4].

In 1987, McCrae and Costa, validated a five factor model of personality for future research and assessment [5]. The authors identified a “Big Five” pattern of personality traits. Of these five personality traits a clear pattern emerged for increased overall risk propensity and saw the combination of the domains of high extraversion and openness supplying the motivational force for risk taking, with low neuroticism and agreeableness providing the insulation against feelings of guilt, and low conscientiousness facilitating overcoming the cognitive barriers of need for control [6].

Risk taking propensity in individuals has been studied [7] and there was heightened interest in scoring instruments, for application in the financial sector, following the collapse of Barings Bank in 1996. The validated and detailed domain‐specific risk taking scale (DOSPERT) assessed five content domains [6]. This was condensed into a compact risk taking index (RTI) and reflected past and present behaviours over six domains. Scores range from 12 to 60, with increasing scores indicating increased risk‐taking propensity. The authors found that risk‐taking propensity has clear links with age and sex, and with objective measures of career‐specific risk taking. Risk propensity was found to be strongly rooted in personality. The RTI was developed as a concise measure with high face validity and answered on a five‐point Likert scale [6].

Patient preference is critical when making clinical decisions, yet research has shown that patients and surgeons often have diverging opinions on their treatment in gastrointestinal conditions [8, 9]. A physician study found that 40% of clinical decisions are made by doctors without their patient's input [10].

The decision to prophylactically defunction a low pelvic anastomosis is controversial and currently a subject of debate in the literature [11, 12]. The ileostomy and its subsequent reversal carry risk in itself and geographical variation in the practice of defunctioning has been observed [13]. This topic is ideal to study as this complex decision is influenced by surgical, patient, situational and personality factors.

Whilst the personality factors of surgeons that affect their decisions are clearly important and relevant [14], this study aimed to elucidate the nurse, surgeon and patient preferences for temporary diverting ileostomies (TDI) and evaluate any discrepancies through a series of three clinical scenarios and delivered by standardised, supervised participant interview. The preferences were ascertained by the validated prospective measures of preference: standard gamble (PMPsg) and time trade off (PMPtto) instruments. These instruments ask the individual to consider a future event and how much of their remaining life expectancy they would be prepared to trade or gamble, if at all, to avoid that future event. These preferences are then related to participants' risk taking propensity as scored with the concise and validated RTI score [8]. The risk taking propensity is a personal characteristic and not a personality trait and this topic was chosen to expand research in this area.

The primary aim of this study was to evaluate nurse, surgeon and patient preferences for diverting ileostomy when undergoing rectal cancer surgery. Secondary aims included: (a) How is this preference affected by risk taking propensity? (b) How much of their remaining life expectancy they would be prepared to gamble or trade to avoid a stoma in several scenarios? (c) To establish at what risk of an anastomotic leak (AL) a surgical nurse, colorectal surgeon and patient would consider would be reasonable to assign a temporary, diverting ileostomy (TDI) to all patients.

## METHODS

A cross‐sectional study involving a one‐off interview and questionnaire assessing how risk taking propensity affects patient and health care worker preferences for a TDI, after rectal cancer surgery, was performed. Participants were categorised into three study groups. Inclusion criteria for Group 1 included stomal therapy nurses or nurses on the colorectal surgical wards. Inclusion criteria for Group 2 included colorectal surgeons who were members or fellows of the Colorectal Surgical Society of Australia and New Zealand. Inclusion criteria for Group 3 included patients attending the colorectal outpatients' clinic and were over 18 years of age and literate. During February 2020 and April 2021, nurses, colorectal surgeons and patients fitting the eligibility criteria were recruited through hospitals in Brisbane, Australia: the Royal Brisbane and Women's Hospital and St Vincent's Private Hospital Northside. Some colorectal surgeons were recruited from other local hospitals to capture all metropolitan colorectal consultants in Brisbane.

Participant consent was obtained, and ethics approval was granted by the St Vincent's human research and ethics committee: HREC 19/06; and the Royal Brisbane & Women's Hospital Human Research Ethics Committee: LNR/2019/QRBW/51323.

### Participants

Consecutive eligible patients were invited to participate during a regular surgical outpatients' clinic appointment. Surgeons and nurses were invited to participate at a convenient time.

All participants were provided with a participant information script (Appendix S1) and were required to provide written consent before inclusion in the study. The researcher conducting the interview assured the participant that scenarios presented in the study were hypothetical situations. The interview included the presentation of background information to participants, collection of demographic data, completion of the RTI, a questionnaire measuring preferences, an assessment of their acceptable risk for anastomotic leak and exploration of attitudes towards stomas (Appendices S2–S5). All participants were assigned a unique numeric study ID to ensure confidentiality. The completion of the one‐off questionnaire and interview took approximately 15–20 min to complete.

Potential bias or interpretational confounders were addressed by having one interviewer for all participants who followed the prescribed script (Appendix S1) and administered the same standardised questionnaires (Appendices S2–S5). Questionnaires 2, 3 and 5 asked the participants to consider themselves as the patient but in Appendix S4 “Visual analogue score assessment of acceptable risk for anastomotic leak”, the participant is asked to consider the risk of anastomotic leakage they feel would be reasonable to give a temporary, diverting ileostomy (TDI) to all patients.” This text was designed to capture the surgeons' threshold for AL as well as patients and nurses.

### Scenarios

Three hypothetical scenarios were chosen to evaluate the participants' attitudes and preferences to stomas. The broad range of the scenario circumstances was intended to allow the participant to interrogate their own preferences and biases related to stomas. The first hypothetical scenario evaluated preferences towards avoiding a permanent stoma. Scenarios two and three evaluated the preferences to omitting a temporary stoma in a higher risk (15%) anastomosis and lower risk (5%) anastomosis. The scripts are presented in Appendix S3. The scenarios were piloted by a group of five surgical registrars who were not included in the study.

### Data collection

Data was collected on paper copies. Deidentified data was entered into the Redcap data capture system, which is encrypted, and password protected. Statistical analysis was performed on deidentified data only.

### Sample size

The proposed sample size of 100 was based on a similarly designed and published scientific study which selected a sample size of 100 patients to detect a difference of 0.1–0.2 in mean PMPsg or PMPtto with a power of 0.9 and significance of 0.05 [9].

### Statistical methods and analysis

Group demographics were described. Patients were compared to nurses and surgeons for the analysis.

The percentage of participants willing to risk or trade any of their remaining life years was recorded (Appendix S3). For those willing to gamble or trade any life expectancy, the percentage of their remaining life years gambled or traded was calculated. Life expectancy was derived using data from the Australian Bureau of Statistics and the remaining life years were calculated by subtracting each participant's age [15].

The participants' attitudes towards stomas were discussed and described (Appendix S5).

SPSS statistical package, was used to analyse the results (IBM Corp. Released 2020. IBM SPSS Statistics for Macintosh, Version 27.0. Armonk, NY). The differences in demographic data were analysed with Chi‐square or one‐way ANOVA. For all other parametric data, differences were analysed with independent samples *t*‐test or one‐way ANOVA (if three or more groups). For all other nonparametric data, differences were analysed with Mann–Whitney U test or the Kruskal Wallis test (if three or more groups). Results were presented as mean ± standard deviation (SD) for parametric data and median with interquartile range (IQR25%–IQR75%) for nonparametric data. A *p*‐value of <0.05 was considered statistically significant.

## RESULTS

### Characteristics of the study sample

One hundred and fifty participants met the inclusion criteria and consented to participate. Participants were categorised as 30 (20.0%) surgical nurses, 20 (13.3%) colorectal surgeons and 100 (66.7%) patients. Nurses, surgeons and patients presented significant differences in age, sex, level of education and prior stoma knowledge (Table 1).

**TABLE 1:** Demographics of the three participant groups

Characteristics	Nurses (*n* = 30)	Surgeons (*n* = 20)	Patients (*n* = 100)	*p*‐value
Age	34.5 ± 7.7	41.3 ± 6.8	56.2 ± 15.5	**<0.001**
Sex (male)	2 (6.7%)	15 (75.0%)	59 (59%)	
Highest level of education				
Junior/Senior certificate	–	–	46 (46.0%)	**<0.001**
TAFE/Diploma	4 (13.3%)	–	21 (21.0%)	
Undergraduate degree or higher	26 (86.7%)	20 (100.0%)	33 (33.0%)	
Prior knowledge of stoma				
Nil	–	–	46 (46.0%)	**<0.001**
Family/Friends/Other	–	–	25 (25.0%)	
Previous/current stoma	–	–	29 (29.0%)	
Healthcare worker	30 (100.0%)	20 (100.0%)	–	

*Note*: Data presented as frequency (percentage) or mean ± standard deviation. The bold values indicate statistically significant result at a *p*‐value of <0.05.

### Risk taking index

**FIGURE 1:**
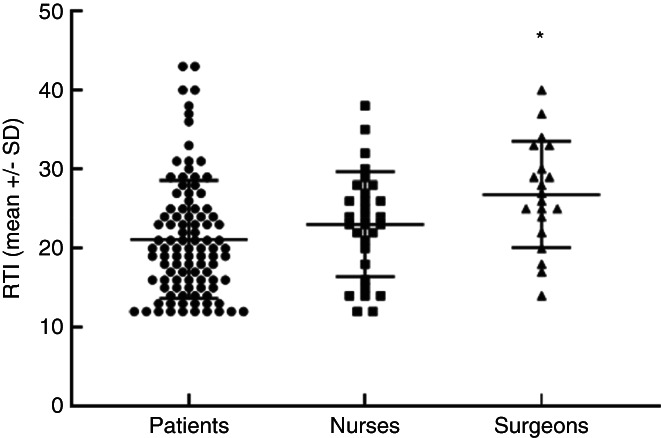
Risk taking index for patients, nurses and surgeons. RTI, risk taking index; RTI ranges from 12 to a maximum of 60, with higher scores indicating a greater risk taking propensity; SD, standard deviation; *p = 0.0002.

The RTI is presented in Appendix S2. Patients (mean ± SD: 20.0 ± 9.8) and nurses (mean ± SD: 23.0 ± 6.6) had a significantly lower RTI when compared to surgeons (mean ± SD: 26.8 ± 6.7; *p* = 0.002) (Figure 1).

### Prospective measures of preference

A summary of the PMPsg and PMPtto results for the three hypothetical scenarios are displayed in Table 2. The strength of the participants desire to gamble or trade is expressed as a percentage of their remaining life expectancy and calculated using data from the Australian Bureau of Statistics [15].

**TABLE 2:** Stoma preferences in three scenarios using prospective measures of preference; standard gamble and time trade off tools

	Nurses (*n* = 30)	Surgeons (*n* = 20)	Patients (*n* = 100)
Scenario 1: *Preference for avoiding a permanent stoma*			
Willing to gamble	4 (13.3%)	9 (45.0%)	38 (38.0%)
*p*‐value	**0.024**		
Percentage of remaining life expectancy willing to gamble	21.8% (8.2%–60.1%)	4.9% (4.2%–11.3%)	31.7% (15.8%–61.4%)
*p*‐value	**<0.001**		
Willing to trade	6 (20.0%)	12 (60.0%)	44 (44.0%)
*p*‐value	**0.012**		
Percentage of remaining life expectancy willing to trade	13.3% (7.5%–35.3%)	7.0% (4.9%–10.7%)	25.2% (13.2%–49.3%)
*p*‐value	**<0.001**		
Scenario 2: *Preference for avoiding a temporary stoma for higher risk anastomosis (15% risk of AL)*			
Willing to gamble	2 (6.7%)	3 (15.0%)	11 (11.0%)
*p*‐value	0.635		
Percentage of remaining life expectancy willing to gamble	51.4% ± 24.7%	4.2% ± 0.8%	15.5% (8.6%–51.0%)
*p*‐value	**0.049**		
Willing to trade	2 (6.7%)	3 (15.0%)	12 (12.0%)
*p*‐value	0.618		
Percentage of remaining life expectancy willing to trade	38.5% ± 6.4%	7.4% ± 3.3%	16.2% (9.6%–26.5%)
*p*‐value	0.063		
Scenario 3: *Preference for avoiding a temporary stoma for lower risk anastomosis (5% risk of AL)*			
Willing to gamble	6 (20.0%)	12 (60.0%)	13 (13.0%)
*p*‐value	**<0.001**		
Percentage of remaining life expectancy willing to gamble	9.3% (4.9%–68.1%)	6.6% (2.7%–12.3%)	18.5% (9.0%–47.1%)
*p*‐value	**0.029**		
Willing to trade	4 (13.3%)	7 (35.0%)	16 (16.0%)
*p*‐value	0.099		
Percentage of remaining life expectancy willing to trade	20.4% (6.1%–40.8%)	4.2% (2.8%–6.5%)	16.2% (6.7%–27.1%)
*p‐*value	**0.033**		

The bold values indicate statistically significant result at a *p*‐value of <0.05.

Abbreviation: AL, anastomotic leak.

Overall, patients were most willing to gamble and trade years of remaining life for the first scenario, pertaining to the permanent stoma, with 38% willing to gamble and 44% willing to trade (Table 2). Nurses were much less willing to gamble and trade years of remaining life to avoid a permanent stoma in Scenario 1 (*p* = 0.024 and *p* = 0.012, respectively). Although surgeons were just as willing to gamble and trade as patients, surgeons gambled and traded fewer remaining years of life than nurses and patients in Scenario 1 (*p* < 0.001).

There was a very similar propensity to gamble and trade between nurses, surgeons and patients in the second scenario, pertaining to the higher risk anastomosis (Table 2). However, nurses gambled significantly more of their remaining years in Scenario 2, gambling 51.4% of their remaining years compared to 4.2% for surgeons and 15.5% for patients (*p* = 0.049).

Surgeons were significantly more willing to gamble and trade life expectancy in the third scenario, pertaining to the lower risk anastomosis, with 60% willing to gamble and 35% willing to trade (Table 2). This compares to nurses, where 20% were willing to gamble and 13.3% willing to trade. For patients, 13% were willing to gamble and 16% willing to trade (*p* < 0.001 for willing to gamble). In this scenario the anastomotic leak rate is 5%, and therefore there is a lesser perceived benefit for a TDI.

### Leak rate to perform a temporary diverting ileostomy

The leak rate to perform temporary diverting ileostomy for surgeons (mean ± SD: 15.0% ± 9.5%) was significantly lower than nurses (mean ± SD: 28.0% ± 32.5%) and patients (mean ± SD: 25.0 ± 30.0; *p* = 0.007) (Appendix S4).

### Attitudes towards stoma

Attitudes towards stomas were very similar across all the analysed groups. The principal concerns for all groups were leakage/unpleasant smells, ability to maintain a romantic relationship, effects on relationship between family and friends and effects on daily function and hobbies. There was much similarity among the groups regarding these principal concerns. However, there were some differences in the groups. Surgeons displayed a higher concern for needing to have further surgery to reverse the stoma. Cultural and religious beliefs were rarely a reason to be concerned about a stoma.

## DISCUSSION

In a hypothetical scenario, surgeons will choose to perform a diverting ileostomy at a perceived anastomotic leak risk of approximately 15.0% and this is significantly different from nurses and patients who would prefer a TDI at a higher leak risk of 28.0 and 25.0%, respectively. This is unexpected as surgeons were found to have a higher risk taking propensity than patients and nurses.

Patients were prepared to gamble and trade significantly more of their remaining life expectancy than nurses and surgeons to avoid a permanent stoma. This may be explained by the familiarity health care workers have with the realities of living with a stoma (Table 2) or the observation that the mean age of patients was higher than nurses and surgeons. This could suggest that older patients would rather risk significant amounts of their remaining life than have a stoma. Additional preoperative strategies may be beneficial to better inform and familiarise patients, and particularly older patients, regarding life with a permanent stoma.

Surgeons would be prepared to gamble significantly less of their remaining life expectancy to avoid a stoma when the AL rate is in the order of 15%. This suggests that the value of defunctioning is held strongly by surgeons at this higher leak rate (Table 2).

Whilst surgeons were more willing to gamble and trade to avoid a TDI in the scenario with a lower AL rate of 5%, they were only prepared to sacrifice a small amount of their remaining life expectancy, and significantly less than patients and nurses. This suggests that defunctioning is held less strongly for surgeons at this lower leak rate (Table 2).

For many patients, anastomotic leak (AL) is an abstract concept, and the majority will have no personal experience of anyone who has experienced an AL. The observation that surgeons have a lower AL threshold than both nurses and patients for a TDI is interesting as nurses and doctors both have personal experience of the consequences and morbidity associated with AL. This is unexpected as surgeons have a significantly higher RTI than nurses and patients. This phenomenon may be explained by a sense of personal responsibility for AL that is borne by surgeons. Additionally, the consequences of AL are managed and seen by the surgeon at every juncture and nurses may not be exposed to all these steps. MacDermaid et al. has also shown that lower surgeon age led to more frequent stoma formation [3].

Studies have investigated the surgeon's personality and how it may affect the heuristic decision to defunction an anastomosis [4]. High levels of the personality trait of openness was identified as an important factor in variation in decision‐making to form a stoma. This study builds on these findings and evaluates the surgeon's risk taking propensity (a personal characteristic) and stoma aversity (also a personal characteristic) alongside nurses and patients.

MacDermid et al. published a study in 2014 concluding that surgeons over 50 years of age and those with a higher propensity for risk taking were less likely to form a stoma across a range of hypothetical scenarios [2]. Moug et al. went on to show that personality traits only influence the next decision if there were other preceding events such as the surgeon receiving recent criticism at a departmental audit meeting [4]. Openness to new experiences here is a desirable personality trait and allows a surgeon to revise their surgical strategy at subsequent operations.

A 2020 systematic review sought to explore common traits in the abdominal surgeons' personality [14]. The authors found that surgeon specific factors influence decision‐making and proposed the Five Factor Model for future research. The international Plato project was introduced and aims to study colorectal surgeons' personalities and risk perception and the influence they may have in several clinical scenarios. The patients' perception of their surgeon's personality has been studied and low levels of emotional stability and conscientiousness were perceived to increase the likelihood of postoperative adverse events [16]. The authors also showed that patients think that risk aversion is a favourable personal characteristic for surgeons. The present study has shown that surgeons are more likely to be risk takers than nurses and patients in their daily lives but are risk averse when it comes to TDI formation.

This study provides important insights into how patients assess risk relating to AL. Patients have expressed a greater tolerance of AL risk to avoid a temporary stoma than surgeons. The aspiration and desired application of this study was that patients could be categorised by their risk taking propensity using the simple and validated RTI tool and that this would allow tailoring of the discretionary use of TDIs. Although one would intuitively expect that patients with a higher RTI, and thus a higher risk taking propensity, would be more likely to wish to avoid a TDI, other characteristics, personality and circumstantial factors may be important. The RTI measures risk taking propensity in the individual's personal lives and may not translate into clinical risk taking. The RTI tool itself may not be sensitive enough to discriminate these confounders and a more comprehensive analysis of surgeons and patients' personalities would provide further insights into other cognitive factors that may influence TDI decisions.

A further limitation of this study is the number of participants in the subgroups and the geographical homogeneity. The population of this study includes nurses, surgeons and patients from the South‐Eastern corner of Queensland, Australia. Studies have shown that rates of TDI formation, among colorectal surgeons, differ across different states and countries [13], whilst other studies have suggested that surgeons from the British Isles, Australia and New Zealand have similar decision‐making heuristics and hence this study's observations may be more widely applicable to these populations [3]. The mean age of patients was significantly greater than surgeons (56.2 ± 15.5 years vs. 41.3 ± 6.8 years) and this may explain some observed differences as risk taking propensity will generally decrease with age. Forty‐six percent of patients had no prior knowledge of stomas in their family or themselves. This may have contributed to the tolerance of higher levels of AL risk expressed and indicate that better stoma information and education would be valuable. Bias related to explanations of the scenarios was limited by the single interviewer supervising all participants.

## CONCLUSION

Surgeons have been shown to have a higher risk taking propensity than patients and nurses but a significantly lower threshold of AL where they would consider a TDI is in the best interest of the patient. This discrepancy may be related to the perception of personal responsibility that a surgeon feels when an AL occurs.

## ACKNOWLEDGMENTS

Open access publishing facilitated by The University of Queensland, as part of the Wiley ‐ The University of Queensland agreement via the Council of Australian University Librarians.

## CONFLICT OF INTEREST

The authors have no conflict of interest to declare.

## ETHICAL APPROVAL

Participant consent was obtained, and ethics approval was granted by the St Vincent's human research and ethics committee: HREC 19/06; and the Royal Brisbane & Women's Hospital Human Research Ethics Committee: LNR/2019/QRBW/51323.

## AUTHOR CONTRIBUTIONS

*Conceptualisation, interviews, data collection, analysis, writing and review*: Ian Mackay. *Conceptualisation, protocol development, analysis, writing, review, submission*: David A. Clark. *Conceptualisation, protocol development, review*: James Nicholson. *Protocol development, review, HREC submission*: Aleks Edmundson. *Conceptualisation, analysis, review*: Daniel Steffens. *Conceptualisation, review*: Michael Solomon.

## Supporting information


Appendix S1‐S5

## Data Availability

The data that support the findings of this study are available on request from the corresponding author. The data are not publicly available due to privacy or ethical restrictions.
