# Supplementary material for: Risk taking propensity: Nurse, surgeon and patient preferences for diverting ileostomy
Source: Colorectal Dis. 2022 May 4;24(9):1073–9. doi: 10.1111/codi.16149 (PMC9790330; doi:10.1111/codi.16149)
Supplement: Supplementary file 1 — Appendix S1‐S5 [file CODI-24-1073-s001.docx]

**Appendix 1:** Participant Information script

A defunctioning ileostomy is a stoma that is surgically created to “de-function” an anastomosis or “surgical join” in the downstream bowel. It is also called a covering ileostomy. The faeces are diverted into a disposable bag attached to the abdomen. They are used when an anastomosis is considered at high risk of not healing or leaking. In some patients, who are at very high risk of leakage, (e.g. those who have had radiotherapy prior to rectal cancer surgery) a surgeon will consider it mandatory that a covering ileostomy is used (i.e. must have one, no choice).

In other cases, a defunctioning ileostomy is used selectively (i.e. it will depend on the individual circumstances of the patient’s health and how the surgery goes). This decision is usually made by the surgeon on the balance of risk. A diverting ileostomy is designed to be reversed and is thus termed a temporary diverting ileostomy (TDI).

Important notes for the decision by the surgeon:

1. A defunctioning stoma, such as a TDI, does not prevent an anastomotic leak; but it does lessen the septic or infective complications of a leak. Patients may still need to return to the operating theatre for further surgery to wash out infection.
2. If a patient suffers an anastomotic leak, and doesn’t have a covering ileostomy, then they will need to return to the operating theatre for surgery to wash out the abdomen and make a stoma (ileostomy or colostomy).
3. Having an ileostomy is not without complication. The inpatient stay is prolonged. There can be complications of obstruction and problems with high output, dehydration and kidney failure. The readmission rate, for these side effects, is around 17% of patients.
4. A further operation is required to close or reverse the ileostomy and there may be complications from this operation.
5. If you do not suffer an anastomotic leak, then, in retrospect, you did not need the ileostomy at all.

Thus, the risks and benefits have to be weighed up in the decision making process. The following questionnaire looks to assess the role of personality and risk-taking behaviour in this decision and how different patients and health care workers might view the outcome.

**What are the pros and cons of a temporary stoma after colorectal cancer surgery?**

**Table: Pros and Cons of temporary stoma**

**A de-functioning or covering ileostomy or stoma does not prevent a leak from a surgical join, but does reduce the risk of infective complications if a leak occurs.**

| **Stoma** | | **No Stoma** | |
| --- | --- | --- | --- |
| **Pros** | Reduce the risk of complications if there is a leak  (this could be lifesaving) | **Pros** | Reduced length of stay |
|  |  |  | No second operation |
|  |  |  | No complications of a stoma |
|  |  |  | Do not have to live with a stoma for 3 months |
| **Cons** | Increased length of stay to learn how to manage a stoma | **Cons** | Increased risk of complications if there is a leak (this could present a risk to life) |
|  | Needs a 2^nd^ operation to reverse with its own risks |  |  |
|  | Complications of a stoma  (such as bowel obstruction or kidney failure) |  |  |
|  | May require readmission (e.g. high output stoma) |  |  |
|  | Reduced quality of life with a stoma for 3 months (e.g. bag leakages at night) |  |  |
|  | Ongoing appointments with stoma nurses |  |  |

Everybody’s individual risk of anastomotic leak is different. This is a short and summarised overview to help participants understand the pros and cons of a temporary covering ileostomy for colorectal cancer surgery.

**Appendix 2:** The Risk-Taking Index

We are interested in assessing your risk-taking behaviours in everyday life. Please could you tell us if any of the following have ever applied to you, *now* or in your adult *past*?

Please use the scale below and circle the most appropriate choice:

1=never, 2=rarely, 3=less often, 4=often, 5=very often

1. How often do you engage in activities that contain recreational risks? (e.g. rock-climbing, scuba diving)

Now In The Past

1 2 3 4 5 1 2 3 4 5

1. How often do you engage in activities that contain health risks? (e.g. smoking, poor diet, high alcohol consumption)

Now In The Past

1 2 3 4 5 1 2 3 4 5

1. How often do you engage in activities that contain career risks? (e.g. quitting a job without another to go to)

Now In The Past

1 2 3 4 5 1 2 3 4 5

1. How often do you engage in activities that contain financial risks? (e.g. gambling, risky investments)

Now In The Past

1 2 3 4 5 1 2 3 4 5

1. How often do you engage in activities that may jeopardize your own safety? (e.g. fast driving, city cycling without a helmet)

Now In The Past

1 2 3 4 5 1 2 3 4 5

1. How often do you engage in activities that contain risks within the social context? (e.g. standing for election, publicly challenging a rule of decision)

Now In The Past

1 2 3 4 5 1 2 3 4 5

**Appendix 3:** Hypothetical Scenario Scripts to measure preference

Please consider the following hypothetical scenarios:

***Scenario 1:***

‘Consider that you are required to have surgery for bowel cancer and the surgeon explains that you require a permanent end colostomy. This means a stoma bag for life with no chance of reversal.’

**(SG 1) Standard Gamble**

‘You are told that there is a hypothetical way to avoid the stoma. Would you be willing to “gamble” any of your remaining life expectancy to avoid having the stoma at all? In this “gamble” you could “win” (and thus not have the stoma), or “lose” (and still have the stoma), if you lose you give up the years of life gambled.

Yes ___

No ___

If yes, how many years of your remaining life are you prepared to gamble?

Please indicate the number of remaining years of your life you are willing to gamble.

Number of years willing to gamble? _______________

**(TTO 1) Time Trade Off**

Are you willing to “trade off” some of your life expectancy to have potential improvement in quality of life by avoiding this permanent stoma? In this “trade off”, you choose how much of your remaining life expectancy you would give up to avoid the outcome. The outcome here is known, but “costs” some of your remaining life expectancy.

Yes ___

No ___

If yes, how many years of your remaining life are you prepared to trade?

Please indicate the number of remaining years of your life you are willing to trade.

Number of years willing to trade? _______________

***Scenario 2: Bowel surgery with a low pelvic anastomosis (surgical join)***

‘Consider that you are having surgery for bowel cancer and that the risk for anastomotic leak is around 15%. In this setting you are being offered a temporary, diverting ileostomy (TDI). The ileostomy would be formed at the time of your surgery and you would go home with this. The stoma would then be closed in approximately three months, if and when the surgical join has healed.’

**(SG 2) Standard Gamble – gambling some your remaining lifespan**

‘Would you be willing to “gamble” any of your remaining life span to avoid having this stoma at all, despite possible complications? In the setting of complications (anastomotic leak), you would need to return to theatre to have the stoma formed. In this “gamble” you could “win” (and thus not have the stoma), or “lose” (and still have the stoma), if you lose you give up the years of life gambled. The outcome is unknown.

Yes ___

No ___

If yes, how many years of your remaining life are you prepared to gamble?

Please indicate the number of remaining years of your life you are willing to gamble.

Number of years willing to gamble? _______________

**(TTO 2) Time Trade Off – trading some of your remaining lifespan, where you will definitely lose that amount of lifespan**

‘As discussed above, you are being offered a temporary, diverting ileostomy (TDI) due to your surgery having a higher risk of anastomotic leak. In this “trade off”, you choose how much of your remaining life expectancy you would give up to avoid the outcome. The outcome here is known, but “costs” some of your remaining life expectancy.

Are you willing to trade any of your remaining life?

Yes ___

No ___

If yes, how many years of your remaining life are you prepared to trade?

Please indicate the number of remaining years of your life you are willing to trade.

Number of years willing to trade? _______________

***Scenario 3: Bowel surgery with a higher pelvic anastomosis (surgical join)***

‘Consider that you are having surgery for bowel cancer and that the risk for anastomotic leak is low or less than 5%. In this setting you are being offered a temporary, diverting ileostomy (TDI). The ileostomy would be formed at the time of your surgery and you would go home with this. The stoma would then be closed in approximately three months, if and when, the surgical join has healed.’

**(SG 3) Standard Gamble – gambling some of your remaining lifespan**

‘Would you be willing to “gamble” any of your remaining life span to avoid having the stoma at all despite possible complications? In the setting of complications (anastomotic leak), you would need to return to theatre to have the stoma formed. In this “gamble” you could “win” (and thus not have the stoma), or “lose” (and still have the stoma), if you lose you give up the years of life gambled. The outcome is unknown.

Yes ___

No ___

If yes, how many years of your remaining life are you prepared to gamble?

Please indicate the number of your remaining years of your life you are willing to gamble.

Number of years willing to gamble? _______________

**(TTO 3) Time Trade Off – trading some of your remaining lifespan, where you will definitely lose that amount of lifespan**

‘As discussed above, you are being offered a temporary, covering ileostomy (TCI) due to your surgery having a low risk of anastomotic leak. In this “trade off”, you choose how much of your remaining life expectancy you would give up to avoid the outcome. The outcome here is known, but “costs” some of your remaining life expectancy.

Are you willing to trade any of your remaining life?

Yes ___

No ___

If yes, how many years of your remaining life are you prepared to trade?

Please indicate the number of remaining years of your life you are willing to trade.

Number of years willing to trade? _______________

**Appendix 4:** Visual Analogue Score Assessment of Acceptable Risk for Anastomotic Leak

With every bowel procedure involving an anastomosis (joining of the remaining bowel after resection of the diseased portion), there is a possibility of an anastomotic leak. Complications of an anastomotic leak may result in a longer hospital stay and further surgery, which will include an emergency temporary stoma.

Surgeons sometimes create a precautionary, temporary diverting ileostomy (TDI) at the initial operation and redirect bowel contents in order to avoid septic and functional complications of an anastomotic leak. However, only a small proportion of these “surgical joins” will actually leak. It is difficult to predict, beforehand, which “joins” will leak.

We are interested in your perspectives regarding at what risk of anastomotic leakage you would consider a temporary diverting ileostomy (TDI) to be ‘worth the risk’.

On the scale below, please indicate with an “x” the risk of anastomotic leakage rate you feel would be reasonable to give a temporary, diverting ileostomy (TDI) to all patients.


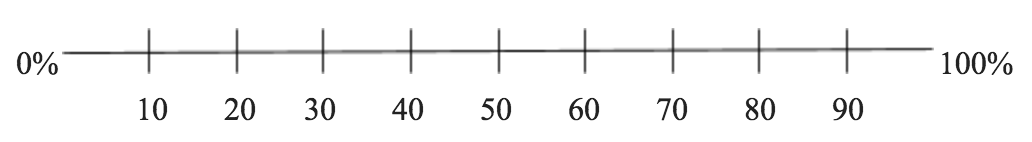


**Appendix 5:** Attitudes Towards Stomas

Past research has shown that patients are stoma averse for various biological, psychological, and social reasons. We are interested in the specific reasons that make a stoma undesirable to patients. Below is a list of major factors that may impact stoma decisions in a negative way. Please rate 1 to 5 with 5 being the most concerning for you.

___ Body Image Concerns

___ Unpleasant previous experience with stoma

___ Effect on relationship with family and friends

___ Social Stigmata

___ Ability to maintain a romantic relationship

___ Leakage, unpleasant smells

___ Cultural and religious reasons

___ Effects on daily function and hobbies

___ Needing to have further surgery to reverse stoma

___ Other (Please Specify) ______________
